# Urban–Rural Differences in Health Care Utilization and COVID-19 Outcomes in Patients With Type 2 Diabetes

**DOI:** 10.5888/pcd19.220015

**Published:** 2022-07-21

**Authors:** Annemarie G. Hirsch, Cara M. Nordberg, Karen Bandeen-Roche, Jonathan Pollak, Melissa N. Poulsen, Katherine A. Moon, Brian S. Schwartz

**Affiliations:** 1 Department of Population Health Sciences, Geisinger, Danville, Pennsylvania; 2 Department of Environmental Health and Engineering, Johns Hopkins Bloomberg School of Public Health, Baltimore, Maryland; 3 Department of Biostatistics, Johns Hopkins Bloomberg School of Public Health, Baltimore, Maryland

## Abstract

**Introduction:**

Two studies in Pennsylvania aimed to determine whether community type and community socioeconomic deprivation (CSD) 1) modified associations between type 2 diabetes (hereinafter, diabetes) and COVID-19 hospitalization outcomes, and 2) influenced health care utilization among individuals with diabetes during the COVID-19 pandemic.

**Methods:**

The hospitalization study evaluated a retrospective cohort of patients hospitalized with COVID-19 through 2020 for COVID-19 outcomes: death, intensive care unit (ICU) admission, mechanical ventilation, elevated D-dimer, and elevated troponin level. We used adjusted logistic regression models, adding interaction terms to evaluate effect modification by community type (township, borough, or city census tract) and CSD. The utilization study included patients with diabetes and a clinical encounter between 2017 and 2020. Autoregressive integrated moving average time-series models evaluated changes in weekly rates of emergency department and outpatient visits, hemoglobin A_1c_ (HbA_1c_) laboratory tests, and antihyperglycemic medication orders from 2018 to 2020.

**Results:**

In the hospitalization study, of 2,751 patients hospitalized for COVID-19, 1,020 had diabetes, which was associated with ICU admission and elevated troponin. Associations did not differ by community type or CSD. In the utilization study, among 93,401 patients with diabetes, utilization measures decreased in March 2020. Utilization increased in July, and then began to stabilize or decline through the end of 2020. Changes in HbA_1c_ tests and medication order trends during the pandemic differed by community type and CSD.

**Conclusion:**

Diabetes was associated with selected outcomes among individuals hospitalized for COVID-19, but these did not differ by community features. Utilization trajectories among individuals with diabetes during the pandemic were influenced by community type and CSD and could be used to identify individuals at risk of gaps in diabetes care.

**Summary**

**What is already known on this topic?**

Studies have reported associations between type 2 diabetes and COVID-19 outcomes, but the extent to which community features modified these associations remains unexplored. Mitigation strategies during the pandemic resulted in type 2 diabetes care disruptions.

**What is added by this report?**

Community features did not modify the associations between type 2 diabetes and severe COVID-19 outcomes among individuals hospitalized for COVID-19. Community features did modify the disruption to type 2 diabetes care during the COVID-19 pandemic.

**What are the implications for public health practice?**

Community features modified the trajectories of disruptions in health care utilization during the COVID-19 pandemic and could be used to identify individuals at risk of gaps in type 2 diabetes care.

## Introduction

Despite early concerns of elevated risk of COVID-19 infection in urban communities (1), studies of infection rates adjusted for socioeconomic factors have shown either no differences or reduced risks between urban and rural areas (2). Urban communities have also been found to have lower risk of severe COVID-19 outcomes (3), attributed to multiple factors, including better access to health care, healthy food, and walkable environments that reduce the risk of severe COVID-19 outcomes (3,4). These same mechanisms may also mitigate risk of severe COVID-19 associated with type 2 diabetes (hereinafter, diabetes).

Reports of associations between diabetes and COVID-19 outcomes have been mixed. Some studies have reported associations with severe COVID-19 (eg, intensive care unit [ICU] admission) and postacute COVID-19 sequelae (5–7), while others have not (8,9). Conversely, most, but not all, studies have reported no association between diabetes and COVID-19 mortality (5,6,9–11). These differences may be due to variation in study design (12) or in study settings from diverse locations around the world. The extent to which community features modify associations between diabetes and COVID-19 outcomes remains unexplored.

The impact of COVID-19 on individuals with diabetes goes beyond COVID-19 infection. Mitigation strategies during the pandemic (eg, suspension of nonurgent care, stay-at-home orders) resulted in diabetes care disruptions (13,14). Health systems serving urban communities have been better able to adapt to COVID-19 mitigation strategies through telehealth technology (15). Thus, gaps in care could be exacerbated in rural communities, which are more likely to have limited access to broadband internet service and greater distances to clinical care settings. The objectives of this study, conducted in geographically diverse communities across 37 Pennsylvania counties, were to determine whether community type and community socioeconomic deprivation (CSD) 1) modified associations between diabetes and COVID-19 hospitalization outcomes, and 2) influenced health care utilization among individuals with diabetes during the COVID-19 pandemic.

## Methods

### Study population and design

We conducted 2 analyses by using electronic health records (EHRs) from Geisinger, a health system serving central and northeastern Pennsylvania. The goal of the first study (hereinafter, the hospitalization study) was to evaluate associations of diabetes and severe COVID-19 outcomes. We conducted a retrospective cohort study of all patients hospitalized with COVID-19 through December 31, 2020, in 10 Geisinger hospitals. The goal of the second study (hereinafter, the utilization study) was to measure the impact of the COVID-19 pandemic on diabetes care. We included all Geisinger patients with diabetes residing in a 37-county region who had at least 1 clinical encounter between 2017 and 2020 (Appendix Figure 1). We identified individuals with diabetes based on encounter diagnoses, diabetes-relevant medication orders, and laboratory test results, as described previously (16).

### Outcomes

The hospitalization study included 5 COVID-19 outcomes: death during hospitalization (up to 120 days after admission) or after hospitalization (up to 220 days) (yes vs no); ICU admission (yes vs no); required mechanical ventilation (yes vs no); D-dimer, a biomarker for thromboembolism (17) (≥0.5 µg/mL vs lower); and troponin level, a biomarker for myocardial damage (18) (elevated vs lower; elevated defined as ≥22 ng/L in men and ≥14 ng/L in women). For the utilization study, we measured weekly rates per 1,000 patients of emergency department (ED) visits, outpatient encounters (including telehealth), hemoglobin A_1c_ (HbA_1c_) tests, and antihyperglycemic medication orders from January 1, 2018, through December 31, 2020. The denominator for the rates included anyone who was alive at the beginning of the measurement year and met diabetes criteria by the end of the measurement year (2018, 2019, or 2020).

### Geocoding and community measurement

We obtained patient addresses from the EHRs and geocoded them to the street level by using ArcGIS World Geocoding Service in ArcMap version 10.4 (Esri) and assigned each patient to an administrative community type based on the residential location. This previously described approach uses boundaries from Pennsylvania’s minor civil divisions and city census tracts to create 3 community types: townships (rural areas to low-density suburbs), boroughs (small towns), and city census tracts, representing a continuum of lower to higher population density (see Appendix) (16). We also classified residential addresses into the US Census Bureau’s categories of urbanized areas, urban clusters, and rural (19).

For each community type, we measured CSD based on 6 indicators from the American Community Survey (2015–2019): percentage of unemployed, with less than a high school education, below poverty level, on public assistance, not in the workforce, and of households without a car (19). A previously described factor analysis demonstrated an adequate model fit of these indicators to a single factor and supported the use of an equally weighted scale based on the sum of the z transformed values of these indicators (20). We have previously reported an association between this CSD measure and diabetes onset (16). We quartiled the index, with the highest quartile representing the most socioeconomically deprived communities.

### Statistical analysis — hospitalization study

The analysis goals were to determine the association between diabetes and each of the 5 COVID-19 outcomes and evaluate whether administrative community type and CSD modified these associations. We first evaluated bivariate associations between individual-level characteristics, administrative community type, and CSD and each of the COVID-19 outcomes. Next, we used logistic regression models with a random intercept for community to estimate the odds ratios (ORs) and 95% CIs for the COVID-19 outcomes.

For each outcome, we evaluated a series of 4 models adjusted sequentially to evaluate potential confounders. We then evaluated effect modification of the association between diabetes and COVID-19 outcomes by CSD and community type by adding cross-products between diabetes and administrative community type or CSD to the models. Global test *P* values were calculated to compare each model with all cross-products to a model with none. The series of models and their covariates are presented below.

Model 1 included age (years; centered linear, quadratic, and cubic terms to allow for nonlinearity), sex (female vs male), race (Black, all other races [Asian, Native Hawaiian or other Pacific Islander, American Indian or Alaska Native] vs White), ethnicity (Hispanic vs non-Hispanic), Medical Assistance, also known as Medicaid in Pennsylvania, as a surrogate for family socioeconomic status (ever vs never) (21), and time period in which hospitalization occurred (early: March to May; middle: June to September; late: October to December 2020). We collapsed races other than White and Black into a single category, all other races, because of small sample sizes. We included time period, based on our hypothesis that COVID-19 outcomes may have improved as the health system learned more about how to manage the disease. In model 2 we added the following comorbid diseases and community features one at a time to both evaluate their associations with COVID-19 outcomes and to determine whether they confounded diabetes associations: chronic kidney disease (vs none); chronic lung disease (vs none); resides in an institutional setting (eg, nursing home) (vs not); administrative community type (borough or city census tract vs township); and CSD (quartiles 2, 3, or 4 vs 1). In model 3 we added both chronic kidney disease and chronic lung disease to model 1 and in model 4 we added institutional setting (nursing homes) to model 3. We conducted sensitivity analyses examining death after discharge and repeated models replacing administrative community type with the urbanicity measure (urbanized areas or urban clusters vs rural).

### Statistical analysis — utilization study

The goal was to determine whether administrative community type or CSD modified the impact of the COVID-19 pandemic on diabetes care. We conducted an interrupted time series analysis for each of the 4 utilization outcomes: weekly rates per 1,000 patients of HbA_1c_ tests, antihyperglycemic medications orders, ED visits, and outpatient (including telehealth) visits. An interrupted time series design measures data at multiple time points before and after the introduction of an intervention, in this case the start of the COVID-19 pandemic in Pennsylvania, to examine the effect of the intervention (22).

We first explored multiple iterations of generalized linear models. After observing high dispersion by using Poisson regression models, we used negative binomial models. We conducted diagnostic checks, including serial residual plots and correlograms, which showed nonnegligible serial autocorrelation. We then added harmonic terms to account for seasonal trends in utilization, but diagnostic checks still showed nonnegligible serial autocorrelation. Thus, we used autoregressive integrated moving average (ARIMA) time-series models of utilization rates to account for the autocorrelation (22).

The study period was January 1, 2018, through December 31, 2020, and the intervention period for the models was from March 16, 2020, through the end of 2020. On March 16, 2020, Geisinger implemented restrictions to elective and nonurgent procedures and Pennsylvania implemented statewide mitigation policies, including an initial stay-at-home order. We added linear splines at time points during the intervention period that we predicted could trigger a change in health care utilization: March 16, 2020; May 4, 2020, when Geisinger reinstated elective and nonurgent procedures; July 13, 2020, which marked an increase in state and national COVID-19 infection rates; and November 30, 2020, when Geisinger reinstated elective procedure restrictions, as ordered by the Pennsylvania Department of Health.

We fit ARIMA models that did and did not account for seasonal utilization trends and models with and without the splines after the start of the intervention period. Based on Bayesian Information Criterion, model fit was better for nonseasonal ARIMA models when the intervention period splines were included; thus, our final models were nonseasonal ARIMA models with 4 linear splines. Data preparation was done by using Stata version 16 (StataCorp LLC). Analyses were performed in R version 4.0.3 (R Core Team).

For each utilization outcome, we evaluated effect modification separately by administrative community type and CSD. Models included a main effect term for each level of the community variable and interaction terms with the following variables: an indicator for the intervention period, study week, and spline terms. ARIMA models allow for 3 parameters: “p,” the number of autoregressive lags incorporated; “d,” the number of past values subtracted (“differenced”) from the current value; and “m,” the number of lags over which errors from prior observations are incorporated in the current error. The auto.arima() function in R’s forecast package (version 8.15) was used to determine the best ARIMA(p,d,q) order for the main effect models for each of the 4 utilization outcomes; d equaled 0 in all cases because no differencing was applied. We then applied the same ARIMA order to the models that evaluated effect modification by administrative community type and CSD. In sensitivity analyses, we repeated the models for each outcome, stratified by the urbanicity measure (urbanized areas or urban clusters vs rural).

## Results

### Hospitalization study

In 2020, 2,751 patients were hospitalized for COVID-19 and 1,020 of these patients met criteria for diabetes before admission (Table 1). Among those hospitalized, 458 died in the hospital and 105 died after discharge. During hospitalization, 650 patients were admitted to the ICU and 342 required mechanical ventilation. Among 2,300 patients with a troponin measure, 1,346 had an elevated level. Among 2,134 patients who had a D-dimer measure, 1,879 had levels ≥0.5 µg/mL.

**Table 1:** Selected Characteristics of Individuals Hospitalized With COVID-19 Through December 31, 2020, by Type 2 Diabetes Status, Pennsylvania^a^

Variable	Type 2 diabetes, n = 1,020 (37.1%)	No type 2 diabetes, n = 1,731 (62.9%)
**Sociodemographics and habits**		
Age at first hospitalization, mean (SD), y	69.9 (13.2)	65.6 (19.4)
Sex, female	467 (45.8)	841 (48.6)
Race, Black	40 (3.9)	89 (5.1)
Hispanic	70 (6.9)	131 (7.6)
Medical Assistance, >0% of time	139 (13.6)	187 (10.8)
Institutionalized housing	144 (14.1)	168 (9.7)
Tobacco use, ever	555 (54.4)	761 (44.0)
**Selected outcomes**		
Died in hospital	198 (19.4)	260 (15.0)
Died after hospital	34 (3.3)	71 (4.1)
Deceased total	232 (22.8)	331 (19.1)
Admitted to intensive care unit	277 (27.2)	373 (21.6)
Required mechanical ventilation	137 (13.4)	205 (11.8)
Hospital readmissions	65 (6.4)	87 (5.0)
**Selected comorbid conditions**		
Chronic kidney disease	417 (40.9)	348 (20.1)
Chronic lung disease	217 (21.3)	229 (13.2)
**Selected laboratory measurements**		
Troponin, plasma, any hospitalization^b^		
Missing	127 (12.5)	324 (18.7)
Elevated level	606 (59.4)	740 (42.8)
Maximum value, mean (SD)	92.6 (441.4)	62.2 (325.8)
D-dimer, plasma, any hospitalization		
Missing	197 (19.3)	420 (24.3)
≥0.5 µg/mL	723 (70.9)	1,156 (66.8)
Maximum value, mean (SD)	3.28 (4.77)	3.02 (4.49)
**Community measures**		
Residential location by administrative community type		
Township	490 (48.0)	891 (51.5)
Borough	302 (29.6)	499 (28.8)
City census tract	228 (22.4)	341 (19.7)
CSD quartiles		
1st (least disadvantaged)	166 (16.3)	337 (19.5)
2nd	202 (19.8)	374 (21.6)
3rd	297 (29.1)	500 (28.9)
4th (most disadvantaged)	355 (34.8)	520 (30.0)

Abbreviation: CSD, community socioeconomic disadvantage.

a Data are shown as n (%) unless otherwise indicated.

b Cut-point for elevated troponin level differs by sex: men, 22 ng/L; women, 14 ng/L.

Diabetes was associated with higher odds of ICU admission and elevated troponin levels in all models, but was not associated with death, mechanical ventilation, or elevated D-dimer levels (Table 2). Chronic kidney disease, chronic lung disease, institutional residence, and age were associated with higher odds of elevated troponin levels and death (Table 2). Age and female sex were also associated with higher odds of elevated troponin levels, while Hispanic ethnicity and hospitalization later in 2020 were associated with lower odds. The only factor associated with mechanical ventilation was the hospitalization time period, with the middle (June–September) and late (October–December) months associated with lower odds of ventilation. No comorbid diseases studied were associated with elevated D-dimer. We found no consistent evidence of effect modification of the associations between diabetes and COVID-19 outcomes by administrative community type or CSD. Administrative community type was not associated with death after discharge and the urbanicity measure was not associated with any of the outcomes (not shown).

**Table 2:** Associations of Type 2 Diabetes Status With 5 Hospitalization Outcomes for COVID-19 Through December 31, 2020, Pennsylvania^a^^,^ b

Variable	Death, total vs not deceased^c^ (n = 2,751)	ICU, any vs not (n = 2,751)	Ventilator, any vs none (n = 2,751)	Troponin ≥22 ng/L vs lower (n = 1,346)	D-dimer ≥0.5 µg/mL vs lower (n = 1,879)
**Model 1: base model**					
Sex, female vs male	0.77 (0.63–0.95)	0.76 (0.63–0.93)	0.85 (0.66–1.10)	1.31 (1.12–1.53)	0.83 (0.71–0.98)
Race and ethnicity					
Non-White vs White	0.56 (0.35–0.89)	0.62 (0.44–0.87)	0.83 (0.55–1.27)	1.02 (0.70–1.49)	0.93 (0.69–1.26)
Hispanic vs non-Hispanic	0.54 (0.28–1.05)	0.79 (0.54–1.14)	1.03 (0.66–1.61)	0.54 (0.35–0.82)	1.06 (0.76–1.47)
Medical Assistance, >0% time vs no time	1.39 (0.95–2.04)	1.02 (0.76–1.37)	0.90 (0.60–1.35)	1.25 (0.90–1.73)	1.28 (0.95–1.71)
Time period for hospitalization (2020)^d^					
Middle months vs early months	0.51 (0.35–0.75)	1.13 (0.82–1.57)	0.35 (0.22–0.55)	0.70 (0.51–0.96)	0.53 (0.39–0.72)
Late months vs early months	0.49 (0.40–0.62)	0.50 (0.40–0.62)	0.43 (0.33–0.56)	0.76 (0.61–0.96)	0.80 (0.63–1.03)
Type 2 diabetes	1.15 (0.94–1.41)	1.21 (1.01–1.45)	1.06 (0.85–1.32)	1.87 (1.56–2.25)	0.96 (0.80–1.15)
**Models 2a–e: adjusted associations of new variables added one at a time to base model**					
2a. Chronic kidney disease vs none	1.46 (1.18–1.80)	1.00 (0.80–1.24)	1.04 (0.84–1.46)	2.60 (2.10–3.23)	0.95 (0.78–1.15)
2b. Chronic lung disease vs none	1.35 (1.06–1.73)	1.10 (0.87–1.38)	1.07 (0.79–1.43)	1.64 (1.29–2.09)	0.99 (0.78–1.25)
2c. Institutionalized vs not	1.63 (1.23–2.16)	1.04 (0.77–1.42)	1.14 (0.77–1.69)	2.23 (1.67–2.97)	0.92 (0.70–1.22)
2d. CSD, 1st vs 4th quartile (least versus most deprived)	0.74 (0.54–1.01)	0.85 (0.64–1.14)	0.96 (0.66–1.39)	0.79 (0.61–1.02)	0.97 (0.73–1.29)
2e. Administrative community type					
Borough vs township	0.92 (0.73–1.15)	1.03 (0.81–1.31)	1.12 (0.85–1.49)	1.18 (0.94–1.48)	0.80 (0.64–1.01)
City census tract vs township	1.05 (0.78–1.40)	0.81 (0.63–1.05)	0.88 (0.65–1.20)	0.96 (0.76–1.21)	0.85 (0.66–1.08)
**Models 3 and 4: fully adjusted diabetes associations** **(model 3: CKD and CLD added to base model; model 4: institutionalized added to Model 3)**					
3. Type 2 diabetes vs none	1.05 (0.85–1.29)	1.21 (1.003–1.45)	1.04 (0.83–1.30)	1.57 (1.30–1.90)	0.97 (0.78–1.16)
4. Type 2 diabetes vs none	1.02 (0.83–1.25)	1.21 (1.0004–1.45)	1.03 (0.82–1.30)	1.54 (1.28–1.86)	0.97 (0.80–1.17)

Abbreviations: CKD, chronic kidney disease; CLD, chronic lung disease; CSD, community socioeconomic deprivation; ICU, intensive care unit.

a Adjusted for age: linear, quadratic, and cubic.

b All data are shown as odds ratio (95% CI).

c Death total = death during and after hospitalization.

d Time period: early (March to May); middle (June to September); late (October to December), all in 2020.

### Utilization study

**Figure 1:**
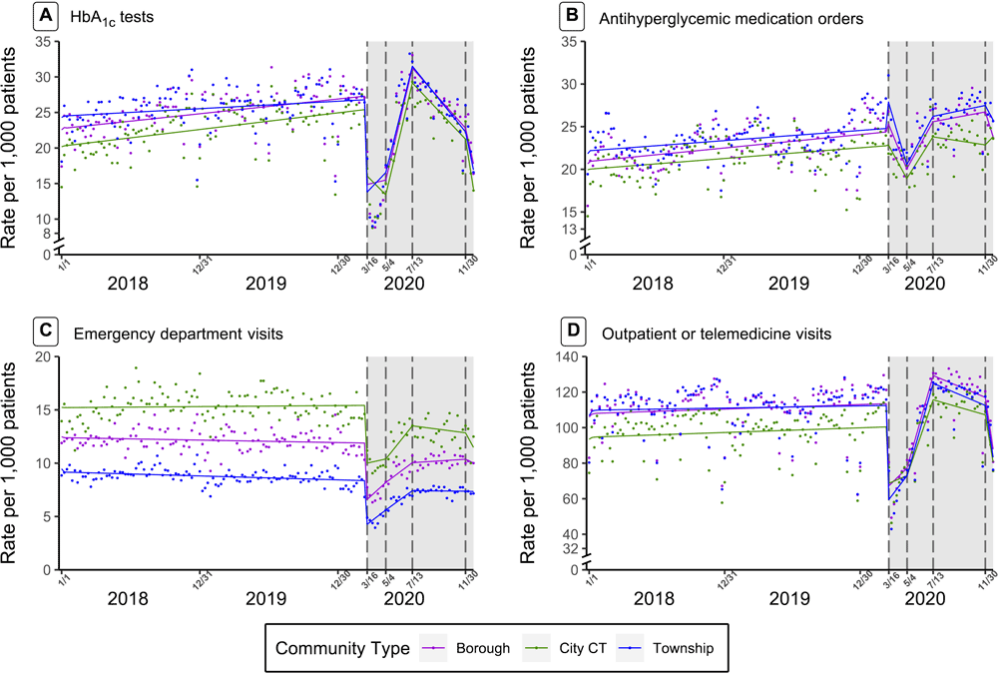
Nonseasonal autoregressive integrated moving average time-series models with linear splines at 4 dates in 2020 (March 16, May 4, July 13, and November 30) of weekly utilization rates per 1,000 patients with type 2 diabetes of hemoglobin A_1c_ (HbA_1c_) tests (A), antihyperglycemic medication orders (B), emergency department visits (C), and outpatient or telehealth visits (D). All plots were stratified by administrative community type. The gray shading indicates the intervention period: March 16, 2020–December 31, 2020.

**Figure 2:**
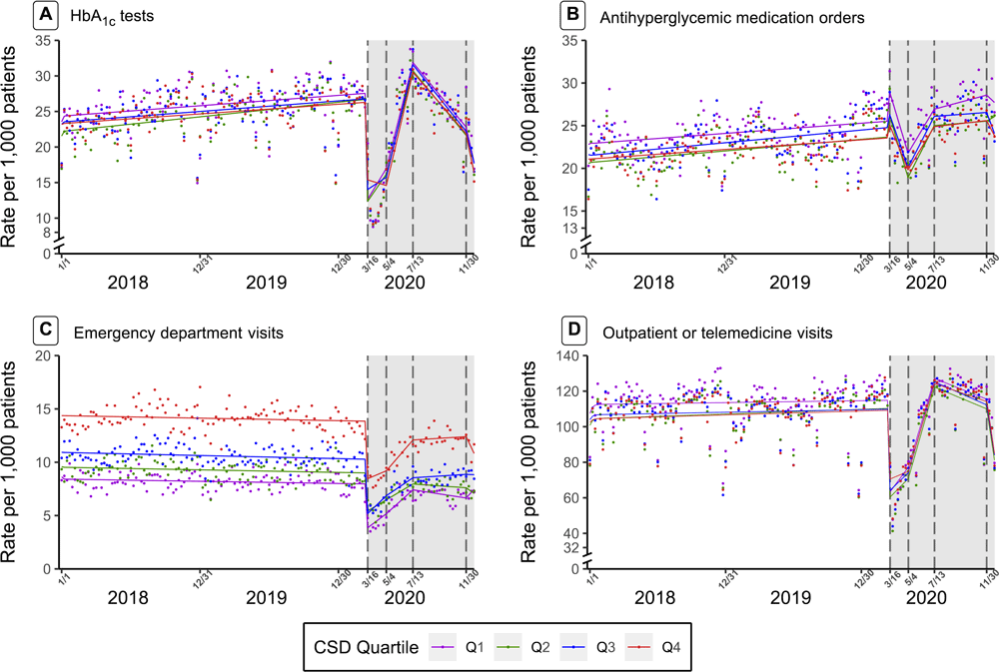
Nonseasonal autoregressive integrated moving average time-series models with linear splines at 4 dates in 2020 (March 16, May 4, July 13, and November 30) of weekly utilization rates per 1,000 patients with type 2 diabetes of hemoglobin A_1c_ (HbA_1c_) tests (A), antihyperglycemic medication orders (B), emergency department visits (C), and outpatient or telehealth visits (D). All plots were stratified by quartile of community socioeconomic deprivation (quartile 4 = most deprived). The gray shading indicates the intervention period: March 16, 2020–December 31, 2020.

A total of 93,401 patients, with a mean age of 57 years, met the criteria for the utilization study (Table 3). Consistent with the demographics of the region that Geisinger serves, individuals were predominately White (93.6%) and the majority resided in townships (55.2%). We present findings based on visual inspection of trends for each outcome for ease of interpretation (Figure 1 and Figure 2). These trends were supported by model coefficients and tests of statistical significance, unless otherwise noted.

**Table 3:** Selected Characteristics of Individuals With Type 2 Diabetes Who Had an Encounter at Geisinger Between 2018 and 2020, by Measurement Year, Pennsylvania^a^^,^ b

Variable	Full cohort (N = 93,401)	2018 cohort (n = 81,393)	2019 cohort (n = 85,812)	2020 cohort (n = 87,612)
**Sociodemographics**				
Age, mean (SD), y	57.0 (14.4)	56.8 (14.2)	56.6 (14.2)	56.4 (14.2)
Sex, female	44,897 (48.1)	39,538 (48.6)	41,507 (48.4)	42,269 (48.2)
Race, non-White	5,993 (6.4)	4,592 (5.6)	5,364 (6.2)	5,822 (6.6)
Hispanic	3,962 (4.2)	3,159 (3.9)	3,606 (4.2)	3,854 (4.4)
Medical assistance, >0% of time	12,898 (13.8)	10,857 (13.3)	11,786 (13.7)	12,390 (14.1)
**Community measures**				
Residential location by administrative community type				
Township	51,564 (55.2)	45,181 (55.5)	47,461 (55.3)	48,337 (55.2)
Borough	27,302 (29.2)	23,800 (29.2)	25,034 (29.2)	25,587 (29.2)
City census tract	14,535 (15.6)	12,412 (15.2)	13,317 (15.5)	13,688 (15.6)
Community socioeconomic deprivation quartiles				
1st (least deprived)	19,634 (21.0)	17,204 (21.1)	18,132 (21.1)	18,478 (21.1)
2nd	21,674 (23.2)	18,941 (23.3)	19,936 (23.2)	20,383 (23.3)
3rd	25,954 (27.8)	22,504 (27.6)	23,757 (27.7)	24,262 (27.7)
4th (most deprived)	26,139 (28.0)	22,744 (27.9)	23,987 (28.0)	24,489 (28.0)

a Individuals were included in measurement year if they were alive at the start of the year and met type 2 diabetes criteria by the end of the year.

b Data are shown as n (%) unless otherwise indicated.

### Administrative community type

Prepandemic rates of ED and outpatient visits differed by community type and urbanicity, such that cities (vs townships) (Figure 1) and urbanized areas and urban clusters (vs rural) (Appendix Figure 2) had higher rates of ED encounters and lower rates of outpatient visits. This disparity persisted throughout the pandemic, as the trajectory of ED and outpatient visits after March 2020 did not differ by administrative community type.

Before the pandemic, weekly rates of HbA_1c_ tests were lower in cities than in townships, but rates were increasing faster in cities than in townships. The drop in weekly HbA_1c_ tests in March was greater in cities than in townships. Statistical output from the ARIMA models indicated that HbA_1c_ tests declined at a faster rate in cities than in townships in March and recovered at a faster rate in cities than in townships in May. However, an inspection of Figure 1A reveals that this finding may be, in part, an artifact of the model, as the nadir in utilization appears to have occurred slightly later than March 16, and hence the modeled results (lines) do not fully reflect the observed data between March and May. All administrative community types experienced the same rate of decline in HbA_1c_ utilization from July through the end of 2020.

Similarly, before the pandemic, weekly rates of antihyperglycemic medication orders were lower in cities than in townships and in urbanized areas than in rural areas. In the week before March 16, there was an increase in the rate of medication orders in townships and boroughs that was not observed in cities, as indicated in Figure 1B by the peaks in rates before the intervention period (shaded gray). In March there was a decline in medication order rates in all administrative community types, but that decline was slower in cities than in townships. Rates started to increase in May, again at a slower rate in cities than in townships. After July, rates continued to increase in townships and boroughs, but started to decrease again in cities.

### Community socioeconomic deprivation

Prepandemic rates of ED and outpatient visits and antihyperglycemic medication orders differed by CSD, such that patients from more deprived communities (quartiles 2, 3, or 4 vs quartile 1) had higher rates of ED encounters, lower rates of outpatient or telemedicine visits, and lower rates of medication orders (Figure 2). These disparities persisted throughout the pandemic, as the trajectory of visits and medication orders did not differ by level of CSD. Statistical output from the ARIMA models indicated that the frequency of HbA_1c_ tests declined at a faster rate in the most deprived community (vs least deprived) in March and recovered at a faster rate in the most deprived community (vs least deprived) in May. However, an inspection of Figure 2A reveals that this finding may be an artifact of the model, as the nadir in utilization appears to have occurred slightly later than March 16, and hence the modeled results (lines) do not fully reflect the observed data between March and May. The rate of decline in HbA_1c_ tests in July through the end of 2020 was the same across levels of CSD.

## Discussion

We evaluated how the COVID-19 pandemic influenced diabetes for both hospitalization outcomes and health care utilization, with a focus on whether these impacts differed by community features. We evaluated 5 hospitalization outcomes (death, ICU admission, ventilator use, elevated troponin levels, and elevated D-dimer levels) and 4 features of health care utilization (HbA_1c_ tests, antihyperglycemic medication orders, ED visits, and outpatient and telehealth visits). We observed that persons with diabetes had higher odds of ICU admission and elevated troponin levels, but these associations were not modified by community features. In contrast, the impacts of the pandemic on the patterns of HbA_1c_ tests and antihyperglycemic medication orders among individuals with diabetes showed important differences by community type, urbanicity, and CSD, providing evidence that clinical care for persons with diabetes during the pandemic was affected by residential setting.

Consistent with prior studies, we observed associations of diabetes with some, but not all, indicators of severe COVID-19 outcomes (5–11). Specifically, patients with diabetes had increased risk of ICU admission and elevated troponin levels. Elevated troponin levels have been associated with mortality among patients with COVID-19, but we did not find an association between diabetes and mortality (18). Elevated troponin among individuals with diabetes may be a marker of existing chronic heart damage rather than damage related to COVID-19 infection (23).

Early in the pandemic, reports from China implicated diabetes as a risk factor for severe COVID-19 outcomes (24). Thus, the elevated risk of ICU admission among persons with diabetes could be due to more severe disease in diabetes or because health systems were more proactively moving individuals with diabetes to ICU settings. Other conditions identified as high risk for poor outcomes early in the pandemic were not associated with ICU admission in our study, providing evidence that ICU admission may have been driven by a need for more intensive care among those with diabetes.

Community type, urbanicity, and CSD were not associated with COVID-19 hospitalization outcomes, nor did they modify associations between diabetes and these outcomes. Prior studies reported that the risk of severe COVID-19 outcomes was reduced in urban communities (2,3) and that the risk of severe COVID-19 was higher in more deprived communities (25) than in the general population. By studying patients hospitalized for COVID-19, our study sample was restricted to those experiencing more severe disease. The mechanisms through which community features influence risk of COVID-19 hospitalization and death in the general population (eg, better access to health care, walkable environments) may have less influence on hospitalization outcomes among those who already have serious disease (ie, are already hospitalized for COVID-19).

Consistent with prior studies (13,14), we observed decreased HbA_1c_ tests, ED and outpatient visits, and antihyperglycemic medication orders at the start of the pandemic, when mitigation measures were implemented at the health system and state level. Utilization was rebounding by May 2020, when mitigation measures were lifted. By July 2020, many mitigation measures had been eased, with all Pennsylvania counties moving to the green phase (lowest risk of infection) on July 3, 2020 (26). Yet we observed that the trend of increasing utilization slowed in July for antihyperglycemic medication orders and ED visits, and for HbA_1c_ tests and outpatient visits, rates started to decline. This could potentially be explained by increased national infection rates starting in mid-July, with cases doubling in 19 US states (27), news that may have influenced local care-seeking behaviors. Thus, individuals with diabetes experienced disruptions in care during multiple phases of the COVID-19 pandemic, including periods of strict mitigation policies and periods of elevated infection rates.

Changes in antihyperglycemic medication order rates differed by community type. The more gradual decline in medication orders in cities may be driven by the peak in medication order rates that occurred in townships and boroughs, but not cities, immediately before the pandemic. The peak in medication orders early in the pandemic has been previously attributed to “panic buying” because of concerns about possible medication shortages (28). Individuals residing in townships and boroughs may have more proactively prepared for a potential disruption in medication supplies, obtaining medications in early March 2020.

ED utilization differences persisted by community type and urbanicity. In contrast with a national report of higher ED utilization in rural, versus urban, communities (29), we found higher ED visits among patients in city census tracts (vs townships) and urbanized areas (vs rural). In prior work in our study region (16), associations between urbanicity and diabetes onset have also differed from national trends, potentially reflecting geographic differences that indicate a need for more localized research on the impact that community features have on health.

This research had numerous strengths. First, our measure of CSD used a spatial scale that is behaviorally relevant, rather than suboptimal scales based on census tract or county boundaries (20). Second, by studying a single health system serving a geographically diverse region, our findings were less vulnerable to confounding by health system factors (eg, treatment protocols) that could differ by community features. 

This study had some limitations. First, the study population was predominately White individuals. Findings may not be generalizable to populations with different sociodemographic characteristics, though findings are likely generalizable to the region studied. Second, patients missing D-dimer or troponin measures were excluded from the analysis of these outcome measures. Third, the administrative community type is challenging to replicate in states without similar municipality boundaries.

In a large, geographically diverse region of Pennsylvania, diabetes was associated with more severe COVID-19 outcomes among individuals hospitalized for COVID-19. These outcomes did not differ by community features, and the higher odds of ICU admission and elevated troponin levels among persons with diabetes was not influenced by community features. Diabetes care was disrupted during periods when COVID-19 mitigation policies were in place and when infection rates were elevated nationally. Community features modified the trajectories of health care utilization during these phases of the pandemic and could be used to identify individuals at risk of gaps in diabetes care. It is important to evaluate the impact of these utilization differences on diabetes outcomes.

## Appendix

**Appendix Figure 1 and Appendix Figure 2** are available at https://www.geisinger.edu/research/departments-and-centers/environmental-health-institute/diabetes.

**Table:** Number and Proportion of Participant Addresses in Administrative Community Types in Each of the US Census Bureau Categories^a^

US Census Bureau categories	Township	Borough	City census tract
Median population density, per square mile	62	1,713	5,537
Rural, n	32,153	2,979	30
Column %	62.4	10.9	0.21
Row %	91.4	8.4	0.09
Urban cluster, n	8,572	9,945	2,948
Column %	16.6	36.4	20.3
Row %	39.9	46.3	13.7
Urbanized area, n	10,839	14,378	11,557
Column %	21.0	52.7	79.5
Row %	29.5	39.0	31.4

^a^ The row percent reflects the percentage of each of the 3 rows: rural, urban, urbanized cluster. Among people living in rural areas, for example, 91.4% live in townships, 8.4% live in boroughs, and 0.09% live in city census tracts. The column percent reflects the percentage of each of the 3 columns: townships, boroughs, city census tracts. In the townships column, for example, 62.4% of townships are rural, 16.6% of townships are in urban clusters, and 21.0% of townships are in urbanized areas.
